# A large-scale genomic investigation of susceptibility to infection and its association with mental disorders in the Danish population

**DOI:** 10.1038/s41398-019-0622-3

**Published:** 2019-11-11

**Authors:** Ron Nudel, Yunpeng Wang, Vivek Appadurai, Andrew J. Schork, Alfonso Buil, Esben Agerbo, Jonas Bybjerg-Grauholm, Anders D. Børglum, Mark J. Daly, Ole Mors, David M. Hougaard, Preben B. Mortensen, Thomas Werge, Merete Nordentoft, Wesley K. Thompson, Michael E. Benros

**Affiliations:** 1 Institute of Biological Psychiatry, Mental Health Centre Sct. Hans, Mental Health Services Copenhagen, Roskilde, Denmark; 2 iPSYCH, The Lundbeck Foundation Initiative for Integrative Psychiatric Research, Aarhus V, Denmark; 3 Norwegian Centre for Mental Disorders Research, KG Jebsen Centre for Psychosis Research, Division of Mental Health and Addiction, Oslo University Hospital, Oslo, Norway; 4 National Center for Register-Based Research, Aarhus University, Aarhus, Denmark; 5 CIRRAU—Center for Integrated Register-based Research, Aarhus University, Aarhus, Denmark; 6 Center for Neonatal Screening, Department for Congenital Disorders, Statens Serum Institut, Copenhagen, Denmark; 7 Department of Biomedicine, Aarhus University and Centre for Integrative Sequencing, iSEQ, Aarhus, Denmark; 8 Aarhus Genome Center, Aarhus, Denmark; 9 Stanley Center for Psychiatric Research, Broad Institute of Harvard and MIT, Cambridge, MA USA; 10 Psychosis Research Unit, Aarhus University Hospital, Risskov, Denmark; 11 Department of Clinical Medicine, Faculty of Health and Medical Sciences, University of Copenhagen, Copenhagen, Denmark; 12 Mental Health Centre Copenhagen, Copenhagen University Hospital, Copenhagen, Denmark; 13 Department of Family Medicine and Public Health, Division of Biostatistics, University of California, San Diego, CA USA

**Keywords:** Diseases, Genetics

## Abstract

Infections and mental disorders are two of the major global disease burdens. While correlations between mental disorders and infections have been reported, the possible genetic links between them have not been assessed in large-scale studies. Moreover, the genetic basis of susceptibility to infection is largely unknown, as large-scale genome-wide association studies of susceptibility to infection have been lacking. We utilized a large Danish population-based sample (*N* = 65,534) linked to nationwide population-based registers to investigate the genetic architecture of susceptibility to infection (heritability estimation, polygenic risk analysis, and a genome-wide association study (GWAS)) and examined its association with mental disorders (comorbidity analysis and genetic correlation). We found strong links between having at least one psychiatric diagnosis and the occurrence of infection (*P* = 2.16 × 10^−208^, OR = 1.72). The SNP heritability of susceptibility to infection ranged from ~2 to ~7% in samples of differing psychiatric diagnosis statuses (suggesting the environment as a major contributor to susceptibility), and polygenic risk scores moderately but significantly explained infection status in an independent sample. We observed a genetic correlation of 0.496 (*P* = 2.17 × 10^−17^) between a diagnosis of infection and a psychiatric diagnosis. While our GWAS did not identify genome-wide significant associations, we found 90 suggestive (*P* ≤ 10^−5^) associations for susceptibility to infection. Our findings suggest a genetic component in susceptibility to infection and indicate that the occurrence of infections in individuals with mental illness may be in part genetically driven.

## Introduction

Infections are one of the major disease burdens and the second leading cause of death worldwide^1^. Infections and inflammation have also been linked to the development of other diseases: autoimmune diseases, cancer, and neuropsychiatric disorders such as schizophrenia and depression^2–6^. Inter-individual differences influence the susceptibility to infection, which is likely to depend on environmental and social factors, vulnerable periods, such as psychological stress or immunocompromised conditions, and the host’s genetic profile^7,8^. Twin and adoption studies as well as epidemiological studies have indicated that hosts’ genetic makeups influence infectious disease occurrences and outcomes of interactions between infectious pathogens and hosts^7–10^. However, the genetic architecture of susceptibility to infection is largely unknown, and knowledge of the genetic composition of infections may help elucidate the mechanism of human complex diseases^8,11^.

Genome-wide association studies (GWAS) have improved our understanding of the genetic basis of common diseases and been used to discover and replicate associations between thousands of genomic variants and hundreds of human diseases^12^. Only a few large-scale studies of the genetics of susceptibility to infection have been conducted, with the largest study being on self-reported common infections and infection-associated procedures^11^. A recent meta-analysis of pooled respiratory infection GWAS (including studies on tuberculosis, influenza, respiratory syncytial virus, SARS-Coronavirus and pneumonia) found only one significant single-nucleotide polymorphism (SNP), in the *IL4* gene^7^. Previous studies have often provided conflicting results and been hampered by low power, differences in study designs, and/or high risk of publication bias^7^. Recently, acknowledging the potential links between psychiatric disorders and infection, studies looking into the genetic of specific infections have been performed, including a study of infection (Toxoplasma gondii, Herpes simplex virus 1, Cytomegalovirus and Human herpesvirus 6) and inflammation in individuals with schizophrenia and bipolar disorder^13^, and a study of Toxoplasma gondii in individuals with schizophrenia^14^ highlighted several genes and/or pathways.

In this largest genetic study of infections requiring hospital contact (hereafter: infections), we utilize a population-based Danish cohort of 65,534 genotyped individuals to conduct a genetic study of overall infection (i.e. a phenotype comprising multiple infection categories, see Methods) from birth to the end of follow-up. The cohort was sampled through the Integrative Psychiatric Research (iPSYCH) initiative^15^, with individuals selected for having a at least one of: autism spectrum disorder (ASD), attention deficit hyperactivity disorder (ADHD), schizophrenia, depression, bipolar disorder and anorexia, as well as a random population sample. We investigate the genetic architecture of susceptibility to infection and examine the link between infections and mental disorders.

## Methods

### Data sources

Data were obtained by linking Danish population-based registers using the unique personal identification number employed in Denmark since 1968^16^. The Danish Neonatal Screening Biobank stores dried blood spots taken 4−7 days after birth from nearly all infants born in Denmark after 1981^16,17^. Information about infections was obtained from the Danish National Hospital Registry, which, since 1977, contains records of all inpatients treated in Danish nonpsychiatric hospitals, and, since 1995, contains information regarding outpatient and emergency room contacts^18^. The Psychiatric Central Research Register covers all psychiatric inpatient facilities since 1969 and outpatient contacts since 1995^19^. Diagnostic information was based on the Eighth Revision of the International Classification of Diseases (ICD-8)^20^ from 1977 to 1993, and ICD-10 from 1994^21^.

### Study sample

All singletons born in Denmark between May 1, 1981 and December 31, 2005 who were residents of Denmark on their first birthday and have a known mother (*N* = 1,472,762) were considered. From this group, 86,189 individuals were included in the iPSYCH2012 sample. Before quality control (QC) our sample included 78,050 successfully genotyped individuals. Following QC, 65,534 individuals remained: 19,645 individuals with no hospital contacts for psychiatric diagnoses (none of ICD-10: F00−F99), and 45,889 individuals with one or more of the following mental disorders: ASD (F84.0-1, F84.5, F84.8-9; *N* = 12,331), ADHD (F90.0; *N* = 14,397), schizophrenia (F20; *N* = 2401), bipolar disorder (F30−F31; *N* = 1391), single and recurrent depressive disorder (F32−F33; *N* = 18,511) and anorexia nervosa (F50.0; *N* = 2551). These diagnoses are based on data from the Danish Psychiatric Central Research Register only. A minority of these individuals (*N* = 1993) have been diagnosed with other psychiatric disorders (other codes in F00−F99) and originally included as part of the random population sample. Individual-based data were available until emigration, death, or 31 December 2012 of a given individual. Three subsets were used across the analyses in this study: only the individuals without psychiatric diagnosis (no F00−F99 diagnosis), only the individuals with psychiatric diagnosis (primary or secondary iPSYCH diagnosis of F00−F99), or a combined sample with a covariate for having a psychiatric diagnosis. More details are given in the relevant sections. With regards to the infection diagnoses, Supplementary Table S1 includes sample sizes for all infection categories. All hospital contacts for infections (both in- and outpatient hospital contacts) were included with ICD-8 and ICD-10 codes listed in Supplementary Table S2, as used in previous studies^4,22,23^, and each person may have a history of more than one infection. We omitted all diagnoses listed as “suspected” or “not found”. Controls for infections were defined as having none of the infection diagnoses in Table S1, and controls for psychiatric disorders were defined as not having any ICD-10 diagnosis of F00−F99 in the Danish Psychiatric Central Research Register, unless stated otherwise.

### Genetic markers, quality control for markers and samples, and imputation

Samples were genotyped on the Illumina Psych chip. Before QC, there were 78,050 samples genotyped in 23 of the original 25 waves. A full description of the procedure of the sample and SNP QC is provided elsewhere^24^. Briefly, a principal component analysis (PCA) was performed using the iPSYCH sample with 1000 Genomes Project samples as a reference panel to compute the initial principal component space. Individuals whose parents and grandparents were born in Denmark were used as a reference in removing individuals who were a certain distance from the multivariate mean of the joint distribution of the first ten PCs. This was then repeated using only the iPSYCH sample to identify subtler within-population differences. Samples were also removed based on genotype missingness, abnormal heterozygosity or ambiguous sex, based on genetic markers. Samples that were identified as duplicates were also removed. Lastly, samples that were found to be related to other samples (first and second degree) were removed, whereby the cases and then samples with a higher genotype call rate were prioritized. Following this, a new PCA was performed to obtain principal components for downstream analyses. For the imputation, only autosomal SNPs were used, and SNPs were removed based on low minor allele frequency (<0.01), Hardy−Weinberg equilibrium *P* value (<10^−6^), having more than two alleles, or being indels. Genotypes were phased with SHAPEIT3^25^ and imputed with IMPUTE2^26^. Imputed markers were removed if they had an INFO score below 0.2, a MAF below 0.001, best-guess genotypes missing in >10% of subjects, HWE *P* < 1 × 10^−6^ (in controls) or a highest probability for a genotype of less than 0.9. Markers were also removed if they were significantly associated with the genotyping wave. Two marker datasets were used: in the GWAS, all post-QC dosage data were used. This dataset included 11,600,722 markers. For the heritability, genetic correlation and PRS analyses, which are based on an aggregation of SNPs, the above dataset was filtered, resulting in a dataset of high-confidence imputations (best-guess genotypes), with markers having an INFO score of at least 0.8 and MAF of at least 0.01. This dataset included 7,071,055 markers. Positions throughout this paper are in hg19.

### Exome-sequencing data

A subset of the iPSYCH sample was exome-sequenced (with an average depth of 20×). Sequencing libraries were produced using a custom adaptation of the Illumina Rapid Target Kit (Illumina ICE Broad Exome) and sequenced on Illumina Hiseqs. The raw reads were mapped using bwa aln (v5.9)^27^ with the parameters -q 5 -l 32 -k 2 to GRCh37, including unplaced and unlocalized contigs and Epstein−Barr (NC_007605.1). PCR duplicates were removed using picard MarkDuplicates, combined per sample, and realigned across indels using GATK IndelRealigner^28^. Variant-calling was performed using GATK’s HaplotypeCaller. Variant filtration was performed using GATK’s variant quality score recalibration modules and the variant annotation was performed using SnpEff^29^. For the analyses that used the exome-sequencing data only, given that we looked only at mutations that may alter the protein coding sequence, we utilized samples even if they were excluded earlier based on relatedness and ancestry, if they passed the other QC measures (*N* = 18,819).

### Statistical analyses

#### Correlation between psychiatric diagnoses and infections

To examine differences in overall infection rates among psychiatric cases and controls, chi-squared statistics were calculated in R^30^. ORs and confidence intervals were calculated using the DescTools package in R^31^. Following this, we performed logistic regressions of the psychiatric diagnosis on the infection diagnosis only in QC-passing individuals from the random population sample, to avoid potential bias resulting from the majority of the sample having been selected for psychiatric disorders. This second analysis used covariates for sex, age and age-squared (to account for nonlinearity with age). The plot for this analysis was exported from Excel with Daniel’s XL Toolbox^32^.

#### SNP heritability

GCTA^33^ (v1.24.7 used with the control subset; v1.91.1 beta otherwise) was used to compute the SNP heritabilities for having any infectious disease. Genetic relationship matrices were calculated for each autosomal chromosome separately with –make-grm and merged with –mgrm. A GREML analysis was then performed with covariates for age, age-squared, sex and the first ten PCs. The analysis was done either with psychiatric controls, psychiatric cases, or the entire sample with an added covariate for having a psychiatric diagnosis.

#### Genetic correlation

A bivariate genetic correlation analysis between having any infection and having any psychiatric diagnosis was run with GCTA v1.91.1 beta, with –reml-bivar and covariates for age, age-squared, sex and the first ten PCs. A Wald test was used to obtain a *P* value for the correlation (assuming no correlation as the null). This was performed in the large sample as well as only with individuals from the random population sample. Additionally, LD score regression v1.0.0 (from August 10 2018)^34,35^ was also used to confirm the genetic correlation, since it is robust to sample overlap^35^. For this purpose, two GWAS were performed using the same SNP dataset as used with GCTA: one GWAS for having any infection (without a covariate for having any psychiatric diagnosis) and another GWAS for having any psychiatric diagnosis (F00−F99 in ICD-10). The above covariates were included in both GWAS. LD scores were calculated with the same SNP dataset using QC-passing samples from the random population sample and a 1 cm window, and a genetic map from 1000 Genomes phase 3. The summary statistics from the two GWAS were QCed with the LD score regression package, after which the genetic correlation was computed.

#### Genome-wide association study

Dosage data were used with PLINK^36^ v1.90b3.34 in a logistic regression model with covariates for age, age-squared, sex, any psychiatric diagnosis, and the first ten PCs. The phenotype used in the GWAS was having any infection.

#### Polygenic risk scores

PRSice^37^ v1.25 was used to calculate polygenic risk scores (PRS). Given that we observe psychiatric patients have a higher incidence of infections, the two variables are not independent^23^. Also, the direction of causality is unknown. To avoid creating PRS that could misrepresent the genetic effects on infection risk (i.e., predicts psychiatric outcomes, which, in turn, predict infection as an environmental exposure) we develop our PRS for infections from a GWAS of infections in patients without psychiatric outcomes (*N* = 19,645). We used the summary statistics from this GWAS as the training dataset, and the subset of psychiatric cases as the target sample (*N* = 45,889). As the PRS was trained in a nonpsychiatric population, any PRS−infection correlations are not likely to be mediated by the current expression of psychiatric outcomes. Furthermore, any confounding between psychiatric diagnoses and infection within the psychiatric population should be independent of the infection PRS by construction and should only contribute to residual variation. In this context, our target sample (the psychiatric cases) will have slightly less power to detect a reduced effect of the PRS relative to a healthy population sample, but the confound should not increase false positives. We used an *r*^2^ threshold of 0.1 in a window of 250 kbp for clumping. As both the training sample and target sample used the same SNP dataset, there was a full overlap between both samples in terms of marker data availability. We ran PRSice with *P* value thresholds of 0.01−1 with intervals of 0.01, and the regression included covariates for age, age-squared, sex and the first ten PCs. From this, we chose the optimal *P* value threshold for downstream analysis.

## Results

In the sample of 65,534 Danish unrelated individuals born after 1981, a total of 28,472 individuals had infections during the study period from birth to end of follow-up. Among the 45,889 individuals with mental disorders, the number was 21,728, and, among the 19,645 individuals with no psychiatric diagnosis, it was 6744.

### Epidemiological correlations

**Fig. 1:**
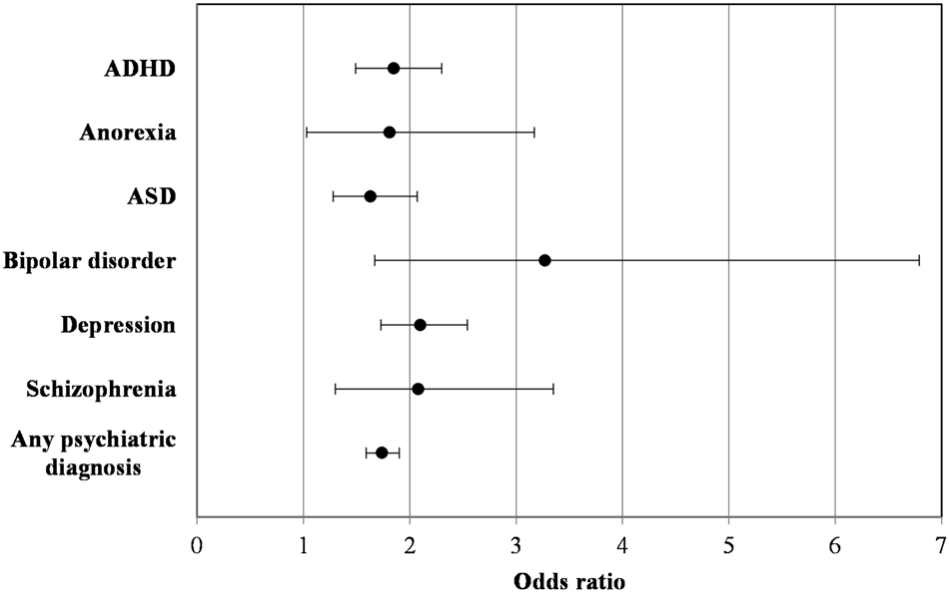
Logistic regressions of psychiatric disorders on infection status

We observed a highly significant correlation between having a psychiatric diagnosis and having an infection (*P* = 2.16 × 10^−208^, OR = 1.72), including highly significant individual correlations between the specific psychiatric diagnoses and infection status (Table 1). To investigate this link further and to account for age and sex, we performed logistic regressions in the random population sample; this avoids potential biases resulting from the selection of cases for the iPSYCH cohort. As can be seen in Fig. 1, the ORs and confidence intervals were greater than 1 for all individual psychiatric disorders and for having any psychiatric diagnosis. All *P* values were <0.05, and all apart from the association with anorexia remained significant after Bonferroni correction. With regards to the individual psychiatric conditions, using controls who do not have any other psychiatric diagnosis in addition to the one in question may lead to biased estimates; we have therefore examined if this is the case here, but the observed effects obtained when defining controls as not having only the diagnosis in question are very similar, as shown in Fig. S1 (the OR for any psychiatric diagnosis is included again as a reference).

**Table 1:** Incidences of psychiatric diagnosis and infection status in our sample

Psychiatric diagnosis	Total number of individuals	Individuals with infections	Individuals without infections	*P* value	Odds ratio (95%CI)
Any psychiatric diagnosis	45,889	21,728	24,161	2.16 × 10^−208^	1.72 (1.66−1.78)
ASD	12,331	5354	6977	7.96 × 10^−60^	1.47 (1.40−1.54)
ADHD	14,397	6730	7667	1.7 × 10^−118^	1.68 (1.61−1.75)
Schizophrenia	2401	1232	1169	4.53 × 10^−60^	2.02 (1.85−2.2)
Bipolar disorder	1391	699	692	3.48 × 10^−33^	1.93 (1.73−2.16)
Depression	18,511	9410	9101	2.83 × 10^−233^	1.98 (1.90−2.06)
Anorexia	2551	1094	1457	1.8 × 10^−17^	1.44 (1.32−1.56)
No psychiatric diagnosis (reference)	19,645	6744	12,901	NA	1.00

### Heritability analysis

**Fig. 2:**
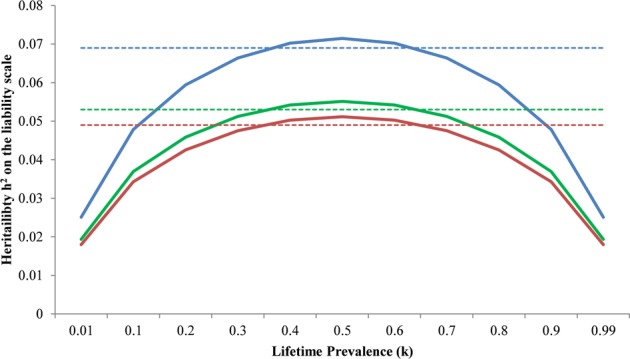
Transformation of the observed heritability of acquiring infection as a function of the lifetime prevalence, *k*. Color coding is blue for iPSYCH psychiatric controls, green for iPSYCH psychiatric cases, and red for the combined sample (with a covariate for any psychiatric diagnosis). Dashed lines show the heritability as estimated using the prevalence in each group when *k* is estimated from the random population sample

We estimated the SNP heritability for overall susceptibility to infection to be 4% (*P* = 0.0018, SE = 0.015) on the observed scale among individuals with no psychiatric diagnosis, and it was 3.5% (*P* = 4.6 × 10^−8^, SE = 0.007) among individuals with a psychiatric diagnosis. These estimates are not significantly different from each other, as can be shown through a *Z* test^38^ (*Z* = 0.358, two-sided *P* = 0.72). The heritability was estimated to be 3.2% (*P* = 1.3 × 10^−12^, SE = 0.005) in the combined sample. To transform the observed heritabilities to liability-scale heritabilities^39^, we adjusted for the proportion of infection cases in the different groups as well as for different values of the lifetime prevalence, *k* (the iPSYCH individuals are too young to estimate the true value of *k*), which allowed us to provide the maximal bound for the heritabilities, given the proportions of cases in our sample. The maximal values of the heritability on the liability scale are around 7% in psychiatric controls, 5.5% in psychiatric cases and 5% in the combined sample (Fig. 2), suggesting a modest genetic component for susceptibility to infection. Figure 2 also shows the heritabilities for a *k* equal to the prevalence in the QC-passing random population sample, which is the minimal lifetime prevalence in our study.

### Genetic correlation

The genetic correlation, *r*_g_, between having any infection diagnosis and having any psychiatric diagnosis was 0.496 (*P* = 2.17 × 10^−17^, SE = 0.058), where the separate heritabilities (on the observed scale) as estimated in this analysis were 3.8% (SE = 0.005) for infection, and 12.3% (SE = 0.006) for psychiatric diagnosis. Since both phenotypes were derived from the same sample, and to avoid any potential ascertainment bias due to the high correlation between them (and given that most individuals in the sample were selected for having a psychiatric diagnosis), the genetic correlation analysis was repeated using only QC-passing samples selected as part of the random population sample (*N* = 21,706: 1062 individuals with both phenotypes; 6693 individuals with only infections; 1113 individuals with only psychiatric diagnoses; 12,838 individuals with neither phenotype). While this analysis had a reduced sample size due to the lower number of psychiatric cases in particular, the observed correlation was similar (*r*_g_ = 0.518, SE = 0.208). Additionally, we confirmed the genetic correlation using LD score regression, which is robust to sample overlap. The *r*_g_ obtained from this analysis was 0.407 (*P* = 9.1 × 10^−5^, SE = 0.104). It is lower than the estimate obtained with GCTA (the latter being potentially biased by the sample overlap), but it is still quite large and significant.

### PRS for infection and predictive value for acquiring infections

**Fig. 3:**
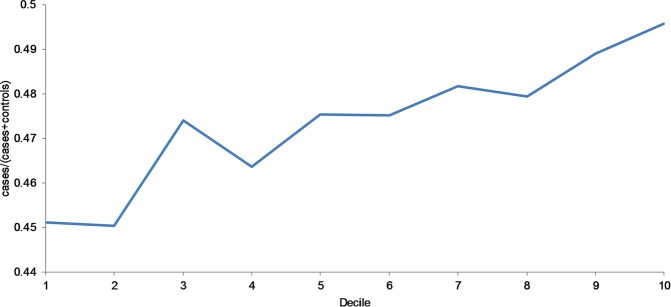
Proportion of cases of infectious diseases at best-fit PRS

The PRS for infection explained a small proportion of the variance in the target sample. We used 100 *P* value thresholds (pT) from the discovery GWAS (0.01−1). After Bonferroni correction, all Nagelkerke *R*^2^’s resulting from *P* value thresholds of 0.04 or higher remain significant at an overall 5% level. The best-fit PRS was with pT = 0.26, resulting in a Nagelkerke *R*^2^ of ~0.9% (*P* = 2.55 × 10^−8^). We additionally examined the change in the proportion of infection cases across PRS deciles with the best-fit PRS. We found that there was an overall trend of an increased proportion of cases, with the difference between the first and tenth deciles being ~5% (Fig. 3).

### GWAS and exome-sequencing

**Fig. 4:**
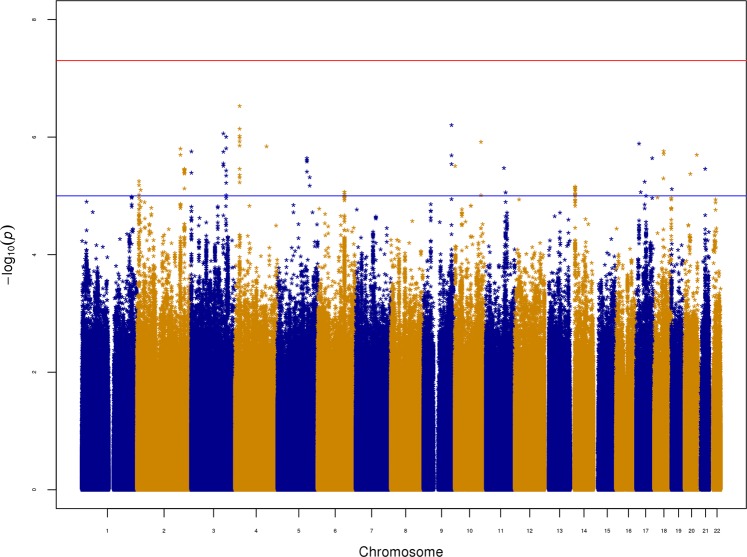
Manhattan plot for the GWAS for overall infection in the combined sample. The red line on the Manhattan plot represents the genome-wide significance threshold (5 × 10^−8^), and the blue line represents the suggestive association threshold (10^−5^). The plot was generated with the “qqman” R script by Stephen Turner and Daniel Capurso

No loci reached genome-wide significance (Fig. 4). Supplementary Figure S2 is the accompanying QQ plot. Overall, there were 90 suggestive associations with *P* ≤ 10^−5^ (Supplementary Table S3), and the top SNP was rs6447952 (*P* = 2.98 × 10^−7^, OR = 0.94 relative to the A allele), which is an intronic SNP in the *SLIT2* gene. Given this gene’s role in both immunity and neurodevelopment and recent findings from several studies concerning disease-risk genes harboring both rare and common variants influencing disease-related traits^40^, we screened a subset of our sample which was exome-sequenced, for potentially deleterious mutations in this gene. We considered frameshift indels, splice-site-, missense- and nonsense-variants (hereafter referred to simply as mutations). We identified 891 *SLIT2* mutation carriers, of whom 426 had at least one infection diagnosis (47.8%). 17,928 individuals did not carry a mutation in *SLIT2*, and 8214 of those had at least one infection diagnosis (45.8%). A hypergeometric test did not find a significant enrichment of individuals with infections among mutation carriers (*P* = 0.117).

## Discussion

In this population-based study we investigated the genetic architecture of susceptibility to infection among 65,534 unrelated Danish individuals. Psychiatric diagnoses were strongly linked with the occurrence of infections, an effect which was observed also in regression models which used a random sample of the Danish population. We found that common genetic variation significantly explained risk of susceptibility to overall infection, to varying degrees, based on psychiatric diagnosis status. In a recent study of the iPSYCH sample, most iPSYCH disorders as well as a cross-disorder phenotype showed significant heritabilities^24^. Given this and the observed heritability for infection, we also examined the genetic correlation between having an infection diagnosis and having a psychiatric diagnosis. We found a strong genetic correlation between the two. Additionally, the phenotype captured by infection requiring hospitalizations showed a polygenic pattern that significantly explained some of the risk for overall infection in an independent sample, albeit to a modest degree.

Differences in host genetics likely influence the host’s susceptibility to infection. However, the combined results of the heritability estimates, the PRS analysis, and the chi-squared and regression analyses showing significant differences in the rate of infection among psychiatric cases and controls may suggest that the environmental component may play a bigger role in acquiring infections. The observed modest genetic contribution to infection indicates that it is primarily nongenetic influences at work, which is not surprising, as infections are most often transient and are transmitted from one person to another. Nonetheless, we found that the polygenic risk score for infection could explain a small proportion of the variance in terms of the risk of having an infection in an independent sample, which is a novel finding.

The heritability estimate for overall infection in the combined sample was lower and more significant than in the two separate groups of psychiatric cases and controls. Thus, adding psychiatric cases to the analysis (while introducing a covariate for having a psychiatric diagnosis) increased the sample size but reduced the heritability estimate. In the individual groups, the heritability was higher among psychiatric controls than among psychiatric cases. This could suggest that, in individuals with a psychiatric diagnosis, the environment may play a bigger role in susceptibility to infection and/or that there are G × E interactions driving this difference. Similarly, there could be a special burden of infection-psychiatric pleiotropic variants in psychiatric cases. It should above all be emphasized that the observed-scale heritability in the two groups did not differ significantly in our study, and, while the question of what is behind this difference is extremely interesting, this study cannot make an authoritative statement on the subject in its current design. It should also be noted that, given the individuals may be diagnosed with infections later on, the lifetime risk estimate *k*, employed in the transformation to the liability scale (calculated from the random population sample), may change, and, as our phenotype encompasses many ICD-10 codes, there are no past epidemiological studies providing an estimate of lifetime risk. As we have examined the heritability for several values of *k*, we believe our results provide a sensible range for the heritability to susceptibility to infection. However, as the true proportion of cases may also change, as the sample ages, these figures could change as well. That said, the SNP heritability estimates we found were comparable to those reported for common infections and infection-associated procedures in a recent study relying on self-reported data^11^.

We observed an increased prevalence of infections among individuals with mental disorders in our random population sample, as well as a high degree of genetic correlation between the two phenotypes, suggesting that, at least, to some degree, this epidemiological observation could be explained by considering the contribution from shared polygenic factors. However, we have shown in a previous study that the robust PRS for schizophrenia does not predict the risk of acquiring infections^23^. The observed genetic correlation could indicate a causal overlap and an etiological role for infections in subgroups of mental disorders. It might also be the case that infections and immune responses could have a triggering role in the development of some mental disorders, with long-lasting subsequent immune alterations in individuals with mental disorders, as is suspected in autoimmune diseases, where infections and susceptibility genes are considered the main risk factors^41^. Moreover, socioeconomic and educational factors might prove important, together with periods of psychological stress or altered behavior, in making the individual more susceptible to acquiring infections. The genetic correlation between overall infection and mental illness, together with the difference in the heritabilities of the two traits, may suggest that the same risk-inducing variants may have a bigger effect on mental illness than on susceptibility to infection.

The GWAS did not identify genome-wide significant SNPs; however, there were 90 suggestive SNPs. The top SNP was rs6447952, an intronic SNP within *SLIT2*. Interestingly, this gene has been reported to be involved in immune response, namely, the SLIT2 protein inhibited leukocyte chemotaxis^42^. Furthermore, this gene is also involved in brain development through neuronal migration^43^. As our GWAS controlled for psychiatric diagnosis, this could be an interesting illustration of a complex genetic factor, perhaps exhibiting pleiotropic effects and/or interactions with other factors. More recently, this gene has been highlighted in studies of schizophrenia and specific infections: Cytomegalovirus and Toxoplasma gondii, although the associations with SNPs in this gene were not genome-wide significant^14,44^. Thus, while no single SNP reached genome-wide significance in our study, possibly due to small effect sizes and a not-large-enough sample size, our results might still be informative regarding potential candidate genes for infection, and for studying the genetic overlaps between infection and mental disorders. However, further studies and functional work are required to support *SLIT2* as a candidate gene for infection, given its only-suggestive association in this study.

The connection between the immune system and psychiatric disorders has been described in many studies; in the case of the iPSYCH sample, a recent study found links between ASD and intellectual disability and some HLA alleles, while also investigating associations with the main disorders represented in the cohort^45^ (see this study for an outline of previous studies investigating these links). Recently, a study of genetic correlation between autoimmune and infection-related phenotypes and psychiatric disorders reported interesting findings; while only one infection category (childhood ear infection) was included, it was significantly positively correlated with ADHD and neuroticism and showed nominal association with depression and angry temperament^46^. Another recent study suggested that risky sexual behavior and schizophrenia risk might have overlapping genetic bases, thus explaining some of the epidemiological correlation between schizophrenia and HIV infection^47^. These results together with our study suggest a complex genetic picture in relation to the link between infection, immunity and psychiatric/behavioral phenotypes.

### Strengths and limitations

The strengths of this study include the prospective design and the population-based nationwide registers in Denmark, ensuring a large study population where all exposures were recorded independently of the outcome and therefore were not subject to selection or recall bias, with the Danish government health-care system being free of charge. Additionally, studies have shown high validity of both diagnoses with infections and mental disorders in the Danish Registers^48,49^. Our infection phenotype may be considered heterogeneous, when each infection category or even each ICD-10 code could be studied individually. However, in this study we chose to investigate the general genetic pathways for susceptibility to any type of infection, and defining the phenotype as such allowed us to do so while at the same time increasing the power of our analyses, as most of the infection categories include a small number of cases.

One limitation of our study is that the age groups represented in this study are relatively young, and, therefore, the lifetime prevalence of acquiring an infection could not be determined. In the same vein, it is also possible that the genetic factors that manifest susceptibility to infection in later stages of life were not captured here. As our dataset included diagnoses of only psychiatric/neurological conditions, infections, and autoimmune diseases (which are correlated with psychiatric conditions)^50^, we did not have an appropriate third category of diseases within which we could examine the incidence of infections. Furthermore, we could include only individuals with hospital contact for infection; hence, the less severe cases of infections were not included. However, this could also be advantageous, as the investigated phenotypes have been so severe as to require a hospital contact, meaning that they may represent a more defined group. Nonetheless, the potential impact of misclassification in the individuals not recorded with infections could result in the genetic associations in our study representing the severity of infection rather than susceptibility to infection. It should also be noted that our models do not incorporate the temporal component of the association between infections and psychiatric disorders; as our phenotypes were complex, and as they may represent more than one infection and/or psychiatric disorder diagnosed in a given individual, our study does not answer questions pertaining to the directionality of this association. Moreover, the genetic contribution might have been greater if studying individuals with severe infections, where genetic susceptibility to infection might play a larger role. Even though this is the largest GWAS to date on infections requiring hospital contact, the included heterogeneous groups of infections might warrant an even larger sample to display the underlying genetic architecture of the susceptibility to infection using GWAS. Lastly, while using a large sample from a homogenous population is advantageous in that it eliminates certain confounders, especially in terms of genetic variation, the results could prove to be specific to the Danish population, as genetic associations across populations can differ based on the presence of the variant in one population but not others, difference in allele frequencies when it is present in more than one population, as well as differences in disease prevalence and variant effect sizes across populations^51^.

In conclusion, we identified a polygenic architecture for susceptibility to infection, with the PRS significantly explaining some of the risk in an independent sample, and found a modest heritability for susceptibility to infection across independent samples. This study confirms the presence of a genetic risk for acquiring infections and a genetic correlation with mental disorders, as well as the high degree of comorbidity between the two. To this effect, we propose that individuals presenting with psychiatric pathology should also be screened for possible infections. Larger-scale studies are warranted both in relation to more precise infection phenotypes and sample size, which could help elucidate the genetic architecture of infectious diseases further.

## Supplementary information

Figure S1

Figure S2

Table S1

Table S2

Table S3
