# Supplementary figures and images for: A large-scale genomic investigation of susceptibility to infection and its association with mental disorders in the Danish population
Source: Transl Psychiatry. 2019 Nov 11;9:283. doi: 10.1038/s41398-019-0622-3 (PMC6848113; doi:10.1038/s41398-019-0622-3)

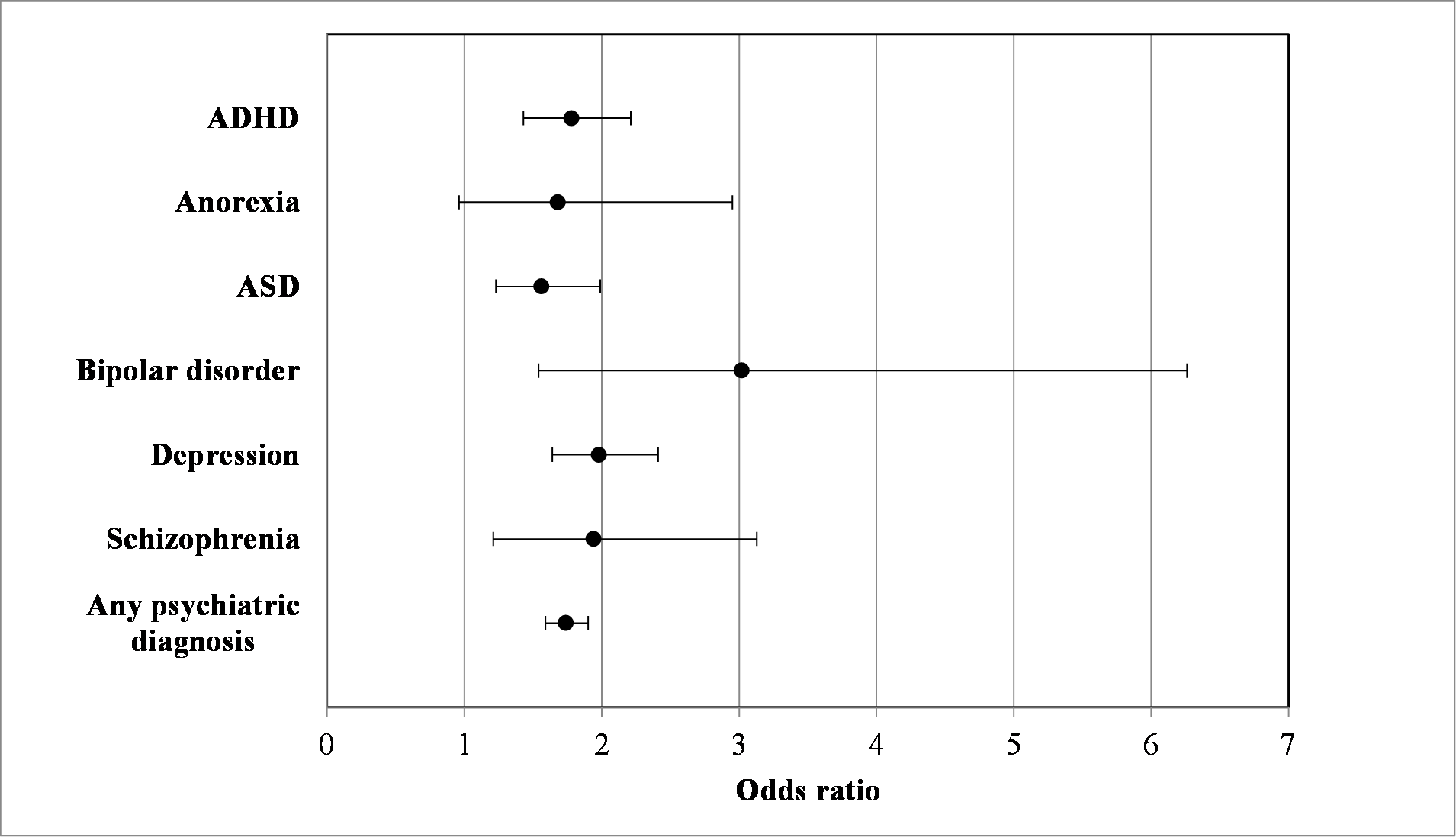

Supplement: Supplementary file 1 — Figure S1 [file 41398_2019_622_MOESM1_ESM.tif]

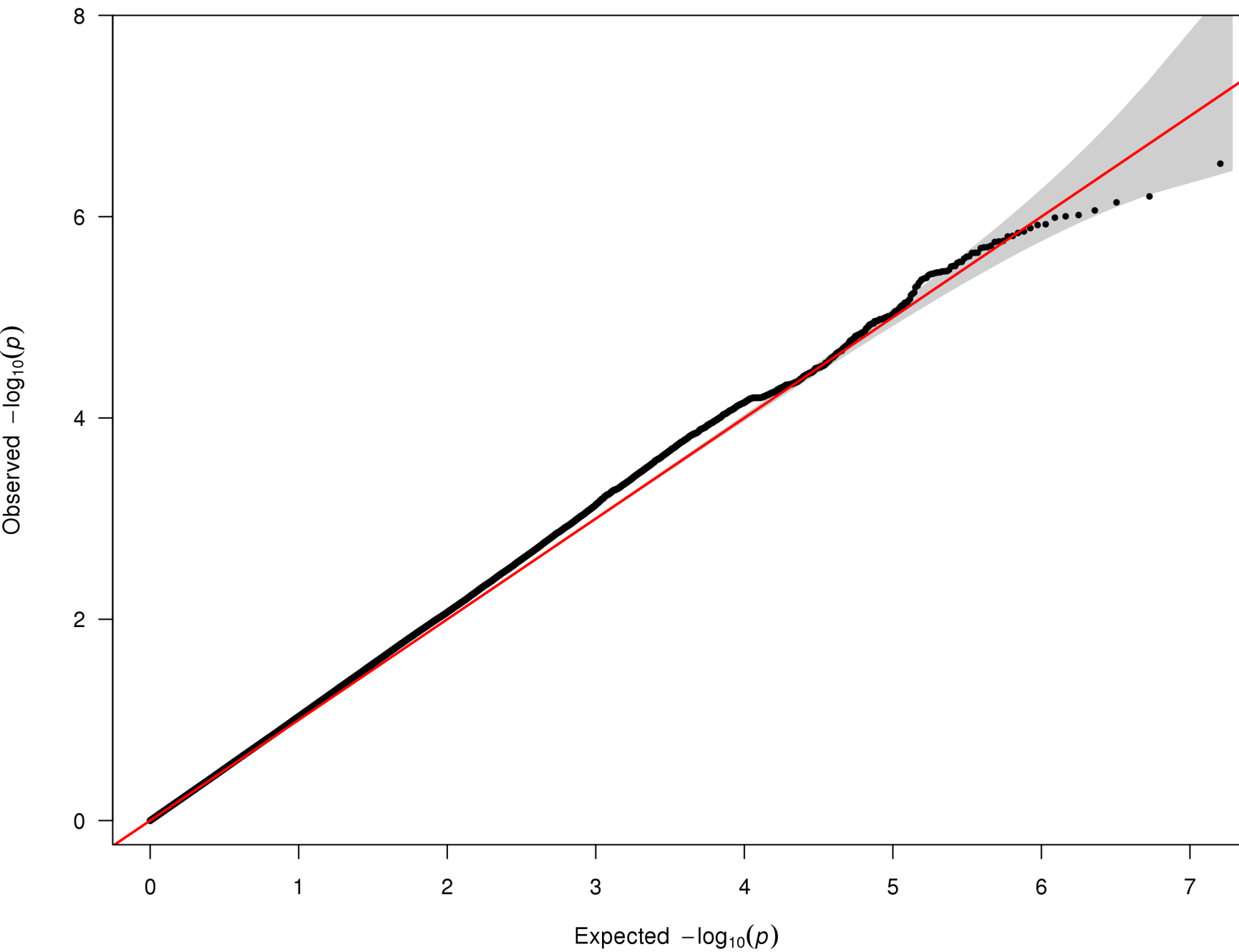

Supplement: Supplementary file 2 — Figure S2 [file 41398_2019_622_MOESM2_ESM.pdf]
