# Supplementary material for: A large-scale genomic investigation of susceptibility to infection and its association with mental disorders in the Danish population
Source: Transl Psychiatry. 2019 Nov 11;9:283. doi: 10.1038/s41398-019-0622-3 (PMC6848113; doi:10.1038/s41398-019-0622-3)
Supplement: Supplementary file 3 — Table S1 [file 41398_2019_622_MOESM3_ESM.docx]

Supplementary Table S1. Sample sizes of individuals with and without infections requiring hospital contacts among 65,534 individuals (45,889 with psychiatric diagnosis and 19,645 without one).

|  | **Entire sample** | | **Individuals without mental disorders** | |
| --- | --- | --- | --- | --- |
| **Disease** | **Number of infection cases** | **Number of controls without infections** | **Number of infection cases** | **Number of controls without infections** |
| **Any infection** | 28,472 | 37,062 | 6,744 | 12,901 |
| **Type of infection:** |  |  |  |  |
| **Bacterial infections** | 11,833 | 37,062 | 2,464 | 12,901 |
| **Viral infections** | 11,914 | 37,062 | 2,799 | 12,901 |
| **Site of infections:** |  |  |  |  |
| **CNS infections** | 551 | 37,062 | 120 | 12,901 |
| **Gastrointestinal**  **infections** | 7,197 | 37,062 | 1,557 | 12,901 |
| **Genital infections** | 749 | 37,062 | 115 | 12,901 |
| **Hepatitis infections** | 111 | 37,062 | 9 | 12,901 |
| **Otitis media infections** | 5,957 | 37,062 | 1,291 | 12,901 |
| **Pregnancy-related   infections** (in the mother while pregnant with the child who is in iPSYCH) | 661 | 37,062 | 103 | 12,901 |
| **Respiratory infections** | 12,958 | 37,062 | 3,010 | 12,901 |
| **Sepsis infections** | 438 | 37,062 | 102 | 12,901 |
| **Skin infections** | 4,709 | 37,062 | 995 | 12,901 |
| **Urological infections** | 2,106 | 37,062 | 386 | 12,901 |
| **Other infections** | 10,203 | 37,062 | 2,213 | 12,901 |
