# Supplementary material for: A large-scale genomic investigation of susceptibility to infection and its association with mental disorders in the Danish population
Source: Transl Psychiatry. 2019 Nov 11;9:283. doi: 10.1038/s41398-019-0622-3 (PMC6848113; doi:10.1038/s41398-019-0622-3)
Supplement: Supplementary file 4 — Table S2 [file 41398_2019_622_MOESM4_ESM.docx]

| **Supplementary Table S2: ICD-8 and ICD-10 codes for site and type of infection** | | |
| --- | --- | --- |
| Infection category | ICD-8 Codes | ICD-10 Codes |
| **Site of infection** | | |
| Sepsis infections | 038 | A40-A41 |
| Hepatitis infections | 070 | B15-B19, K770 |
| Gastrointestinal infections | 000-009, 540 | A00-A09, K35 |
| Skin infections | 035, 050-057, 110-111, 680-686 | A46, B00-B09, L00-L08 |
| Respiratory infections | 460-486 | J00-J18, J22, J36 |
| Urological infections | 580, 590, 595 | N00, N05, N300, N370, N390, N129 |
| Genital infections | 604, 612, 620, 622 | N45, N51.2, N70, N70.0, N70.9, N76.0, N76.2, N76.4, N77.0, N77.1, N518B |
| Pregnancy-related infections | 630, 635, 670 | O23, O26.4, O85–O86, O98 |
| Otitis media infections | 381-382 | H65-H67 |
| Central nervous system infections | 013, 027.01, 036.09, 040–043, 045-046, 052.01, 053.02, 054.03, 055.01, 056.01, 062-065, 071.99, 072.02, 075.01, 079.29, 090.49, 094.9, 320, 322–324,  474 | A02.2C A06.6, A17, A229C, A32.1, A39.0, A50.4, A514B, A521A-B, A521B, A548A, A548D, A80-89, B00.3-B00.4, B01.0- B01.1, B02.0- B02.1, B05.0-B05.1, B06.0, B26.1-B26.2, B37.5, B45.1, B58.2, B60.2, E236A, G00-G07 |
| **Type of infection** | | |
| Bacterial infections | 000-005, 008.09-008.39, 010-018, 020-023, 025-039, 073.99, 076.99, 079.84, 080-083, 088.99-089, 090.09-090.59, 091-098, 100-104, 320.09-320.80, 322, 380-382, 390-392, 420-421, 461- 464.03, 481- 483, 501.99, 508.00-508.03, 510, 513, 522.59, 526.49, 527.22, 528.38-528.39, 529.03, 540.01, 566-577.03, 590, 595.00-595.01, 597.00, 597.03, 599, 601, 604, 607.39, 611.00-611.01, 612, 614, , 620.90, 620.99, 622, 629.49, 630-631.39, 635, 670,678.01, 680, 681.08-683, 684.08-684.09, 685.01, 686.00-686.08, 710, 720.00-720.29, 720.31, 732 | A022C, A03-A05, , A15-A58, A65-A79, B088D, B95-B96 , D733, E060A, E236A E321, , I301A-D, I320, I398, I410, I430, I520A, J01, J020, J030, J13-J15, J160, J170, J851, J86, K61, K67, , K040A, K046A, K052A, , K112A, K113, K122, K130A, K140A, K209A, K351, K650N, K630, K930, L00-L04, L08, M00, M010-M013, M015B, , M463, M490-M492, M680, M725A, N10-N12, N136, N151, N200I, N201I, N300, N341, N390, N410, N412, N431, N450, N459, N510A,C, N511, N70-N74, N764, N980, O23, O753, O85-O86, O980-O982, T814A-D, T814F-J, T793, T802, T874, T880 |
| Viral infections | 008.80, 008.89, 008.90, 040-046, 050-065, 067- 072, 074-075, 078, 079.82, 460,464, 465, 470-480 | A08, A60, A630, A80-A89, A90-A99, B00, B01-B09, B15-B27, B33-B34, B97, G020, G051, H621 A-B, H671 A-B, I400B, I411 A-B, J00, J04-J06, J10-J12, J171, J203-J207, J210, K770A-B, K871A-B, M014-M015, N518B, N770D, N771B, N771G, N771L |
| Other infections | 006-007, 008.99, 009.99, 084, 087, 089, 099.91-099.99, 110-117-131, 136.00, 136.03, 572, 998.59 | A06-A07, A085, A09, A59, A63-A64, B35-60, B64-B83, B87-B89, B99, G02, G040, G049, G052A-C, G052E -J, G079D, H622, I301, I33, I400, I411-412, I521C, J02-J03, J18, J172, J173, J178, J22, J998B-C, K35, K770C-E, K750, L303, M016, M631C-F, M632A, M651, M711, N160D, N370A, O983, O986-O989, T89 |
